# Influence of Notch inhibitor DAPT and ROCK inhibitor Y-27632 on differentiation in nasal cell line CI-pNaEC as an in vitro model for preclinical testing of drugs

**DOI:** 10.1007/s44164-026-00145-7

**Published:** 2026-08-19

**Authors:** Leonie Pia Isabelle Klintz, Stephan Reichl

**Affiliations:** 1 Institute of Pharmaceutical Technology and Biopharmaceutics, Technische Universität Braunschweig, Mendelssohnstraße 1, Brunswick, 38106 Germany; 2 Center of Pharmaceutical Engineering - PVZ, Technische Universität Braunschweig, Franz-Liszt-Str. 35a, Brunswick, 38106 Germany

**Keywords:** Nasal epithelial cell line, Nasal mucosa, In vitro model, Mucus, Ciliated cells, Notch inhibition, ROCK inhibition

## Abstract

In the preclinical testing of novel drugs cell-based in vitro models can be used to replace animal testing. The cell line CI-pNaEC is a promising candidate for a suitable in vitro model of the nasal epithelium but suffers from low ciliation and inconsistent epithelial architecture. In this study, the influence of Notch and ROCK inhibition on differentiation benchmarks in CI-pNaEC was investigated. Ciliation was monitored via immunofluorescence for acetylated α-tubulin, mucus expression was visualized and quantified using alcian blue, barrier properties were assessed using TEER measurements and permeation studies, while histology was evaluated by light microscopy. Treatment with low-micromolar concentrations of DAPT markedly increased the proportion of ciliated cells and promoted a more uniform, pseudostratified epithelial layer, while mucus secretion remained comparable to untreated controls. However, DAPT also induced a slight increase in barrier properties, resulting in permeation coefficients below ex vivo nasal tissue benchmarks for some markers. In contrast, Y-27632 alone had a visible effect on ciliation and histological uniformity, with minimal effects on mucus expression, and its combination with DAPT did not further improve differentiation. The results demonstrate that targeted Notch inhibition at low doses significantly enhances ciliation and epithelial organization in CI-pNaEC without compromising mucin production, although barrier function is modestly affected.

**Supplementary Information:**

The online version contains supplementary material available at https://doi.org/10.1007/s44164-026-00145-7.

## Introduction

Nasal drug delivery has gained considerable attention as a non-invasive route for administering pharmaceutically challenging agents [1]. The highly vascularized nasal mucosa allows for rapid systemic uptake while bypassing both the gastrointestinal tract and hepatic first-pass metabolism. As a result, there is growing interest in formulating drugs specifically for intranasal administration [2, 3]. Thus, a wide array of complex therapeutics from fentanyl, over peptide hormones such as glucagon and insulin and other pharmaceutically challenging drugs can be delivered painlessly with high bioavailability via the nasal application [4, 5]. Despite these advantages, mucociliary clearance remains a major challenge for effective intranasal delivery [6, 7]. Goblet cells secrete mucins, that form a viscoelastic mucus gel (95% water, 2–5% mucins) that protects the epithelium but can trap drug carriers within its three-dimensional mesh architecture [7–9]. Coordinated beating of motile cilia, which are hair-like tubulin extensions on respiratory epithelial cells, transports this mucus toward the nasopharynx, thereby clearing mucus and any entrapped drugs from the nasal passages [7, 10, 11].

Although animal models are needed in preclinical drug development, the high variability, elevated costs, ethical concerns, and limited translational relevance remain a major constraint [12, 13]. In line with the 3R principles (replace, reduce, refine), in vitro cell culture systems offer a promising alternative [14–16]. However, to reliably predict in vivo behavior and thus shorten drug development timelines, an in vitro nasal model must faithfully recapitulate native tissue architecture and function. In particular, this includes appropriate barrier properties and in vivo–like epithelial pseudostratified histology of 40–100 µm thickness, as well as functional mucociliary clearance, since the mucus layer constitutes a critical barrier to drug absorption [17, 18]. To date, no established cell model combines all these essential features. The RPMI 2650 cell line displays physiologically relevant barrier characteristics, yet in static culture it neither develops functional motile cilia nor secretes sufficient mucus, making it unable to model mucociliary clearance [18–22]. Conversely, Calu-3 and UNCN3T cells produce mucus yet exhibit limited ciliogenesis and have not been fully characterized in terms of their barrier function [22]. Although induced pluripotent stem cell-derived airway epithelia promise an unlimited cell supply, they require lengthy and costly differentiation protocols, produce inconsistent ciliation, and often display anomalous ion transport [23–25]. Primary cell-based systems, including MucilAir™, more accurately reproduce nasal mucosa features, such as ciliation and mucus production. However, their reliance on donor-derived cells limits subculturing potential, causes inter-donor variability, constrains availability and results in inadequate barrier properties [26, 27]. Thus, a scalable, reproducible airway model that integrates barrier function, mucus production, and ciliary activity remains elusive.

The recently introduced porcine nasal epithelial cell line CI-pNaEC is a promising candidate for a more fitting in vitro model of human nasal mucosa, which secretes in vivo-like mucus and expresses suitable barrier properties [28–30]. However, while ciliation was visible, it remained sparse compared to physiological nasal mucosa. Additionally, a histological evaluation revealed structural alterations in the epithelial tissue, including focal hyperplasia and deviations from the physiological pseudostratified organization in certain regions of the culture [28].

To overcome the limitations of CI-pNaEC, two cell-fate-regulating pathways were studied. Cell fate in nasal epithelial cells is largely controlled by the Notch pathway, which is activated by cell-to-cell contact [31, 32]. In brief, following cell–cell interactions, the Notch intracellular domain is released upon Notch receptor activation involving γ-secretase. It is then transported to the cell nucleus, where it activates genes associated with proliferation, differentiation, and apoptosis [33, 34]. In the nasal mucosa, high levels of Notch signaling direct basal cell differentiation toward mucus-producing goblet cells, whereas reduced Notch activity biases differentiation in favor of ciliated cells [35]. Consequently, Notch inhibition represents a promising strategy to enhance epithelial ciliation by limiting goblet‐cell formation [36–40]. One widely used Notch pathway inhibitor is DAPT, which targets γ-secretase and interrupts the Notch receptor activation cascade [41, 42]. Therefore, DAPT supplementation is a potential approach to shift the differentiation of CI-pNaEC towards multiciliated cells and thereby accelerating ciliogenesis.

The ROCK pathway influences various aspects of cell metabolism and cytoskeleton organization [43, 44]. For instance, in preclinical cancer models, overexpression of ROCK pathway revealed an effect on tumor cell overgrowth by promoting mitosis and inhibiting apoptosis [45]. In contrast, the ROCK pathway appears to play a role in basal body formation during ciliogenesis in multiciliated cells [46]. Therefore, supplementing CI-pNaEC cultures with Y-27632, a ROCK-inhibitor, could attenuate epithelial overgrowth and thereby contribute to a more physiologically accurate pseudostratified architecture. However, any histological improvements must be evaluated in relation to the potential inhibition of ciliogenesis due to impaired basal body formation.

In this study, the impact of the inhibition of Notch and ROCK pathways on the differentiation of CI-pNaEC cultures, with the aim of improving their histological architecture towards pseudostratification and increasing ciliation were systematically assessed. CI-pNaEC cultures were treated at the air–liquid interface with varying concentrations and combinations of the γ-secretase inhibitor DAPT and the ROCK inhibitor Y-27632. The resulting histological architecture, ciliation density and cilia length, mucus production and epithelial barrier integrity were evaluated. Based on these findings, an improved differentiation protocol for CI-pNaEC can be established that more closely mimics the physiological nasal epithelium, particularly in terms of robust ciliated cell formation and pseudostratification.

## Materials and methods

### Cell culture models

CI-pNaEC (InSCREENeX, Germany) is a novel *p*orcine *na*sal *e*pithelial *c*ell line that was established recently by lentiviral transduction using the CI-SCREEN gene library [29, 30, 47]. CI-pNaEC cells were maintained in huNASA medium (Inscreenex, Germany) supplemented with 1% antibiotics (Sigma Aldrich, USA) with medium changes every 2–3 days. For experiments, 2.5·10^5^ cells were seeded onto 12-well PET Inserts (0.4 µm pore size; Greiner Bio-One, Germany) and cultivated submerged in huNASA for 3 days.

When air–liquid interface (ALI) was established on day 3, cells were randomly introduced to one out of 12 huNASA-based media formulations containing DAPT (N-[N-(3,5-Difluorophenacetyl)-L-alanyl]-S-phenylglycine t-butyl ester, MedChemExpress, USA) and Y-27632 (MedChemExpress, USA) in varying concentrations (Table 1). Both DAPT and Y-27632 were pre-dissolved in DMSO, and all media were adjusted to the same final DMSO concentration of 0.0075%. HuNASA medium without any supplementation served as blank control (Y0/D0), whereas medium containing only DMSO was used as the vehicle control. All experiments were conducted after 21 days of ALI culture.

**Table 1:** Media composition for ALI differentiation

Final concentration Y-27632 [µM]	Final concentration DAPT [µM]	Final concentration DMSO [%]	Abbreviation
0	0	0	Y0/D0
5	0	0.0075	Y5/D0
10	0	0.0075	Y10/D0
5	5	0.0075	Y5/D5
5	10	0.0075	Y5/D10
10	5	0.0075	Y10/D5
10	10	0.0075	Y10/D10
0	2.5	0.0075	Y0/D2.5
0	5	0.0075	Y0/D5
0	7.5	0.0075	Y0/D7.5
0	10	0.0075	Y0/D10
0	0	0.0075	vehicle

### Histological sections

Cultures were fixed overnight at 4 °C in formaldehyde (Carl Roth, Germany). Samples were then dehydrated through a graded ethanol series and infiltrated overnight at 4 °C with Technovit® 7100 (Kulzer GmbH, Germany). Embedding was performed using Technovit® 7100 according to the manufacturer’s protocol. Samples were sectioned to a width of 2 µm using a Microm HM 355S microtome (Thermo Scientific, USA), stained with hematoxylin and eosin, and imaged on an Olympus IX 50 inverted microscope equipped with an Olympus XC 30 camera (Olympus, Japan). Epithelial thickness was assessed using ImageJ software.

### Alcian blue staining

Mucus distribution on the inserts was assessed by alcian blue staining. Cultures were fixed with Sainte-Marie’s reagent for 15 min [48]. Then 1% alcian blue (Morphisto, Germany) was applied to the apical chamber for 30 min. Excess stain was removed by two rinses with 3% acetic acid (Carl Roth, Germany). Overview images were acquired on an Olympus IX 50 inverted microscope equipped with an Olympus XC 30 camera.

### Mucin quantification

Mucin was quantified based on a colorimetric method as described before [28, 29]. Briefly, 500 µL of distilled water was added to the apical chamber and the cell layer was gently detached from the PET insert using a silicone scraper. To release cell-bound mucins, samples underwent five wash-and-spin cycles (2540 × g, 3 min) in distilled water. Mucins were then precipitated overnight by adding 1% alcian blue (Morphisto, Germany) and 25 mM MgCl₂ (Acros Organics, Belgium). Excess dye was eliminated through repeated wash-and-spin steps (2540 × g, 10 min) in a mixture of 25% ethanol, 0.1 M acetic acid, and 25 mM MgCl₂ until the supernatant was colorless. The resulting alcian blue-mucin pellet was solubilized in 10% SDS (Roth, Germany), and absorbance was measured at 620 nm using a Tecan Infinite® M Plex reader (Tecan, Switzerland). Mucin concentrations were calculated from a standard curve generated with serial dilutions of porcine gastric mucin III (Sigma-Aldrich, USA).

### Permeability testing

Permeation experiments were performed as described elsewhere [28–30]. In brief, the following marker compounds were used for para- and transcellular transport, as well as for a high molecular weight substance: sodium fluorescein (250 µg/mL), rhodamine B (50 µg/mL), and FITC-Dextran 4000 (500 µg/mL), all of which were obtained from Sigma-Aldrich Chemie GmbH in Germany. Cultures were equilibrated for 30 min at 37 °C in prewarmed Krebs–Ringer buffer (KRB). Barrier integrity was confirmed by measuring transepithelial electrical resistance (TEER) with an Endohm chamber and EVOM^2^ resistance meter (World Precision Instruments, USA). Thereafter, the basolateral compartment was filled with 2 mL fresh KRB, and 0.4 mL of the test solution was applied apically to initiate permeation. At 10, 20, 30, 60, 90, 120, 150 and 180 min, 100 µL samples were withdrawn from the basolateral side and immediately replaced with prewarmed KRB. Marker concentrations were measured on a Tecan Infinite® M Plex reader (Tecan, Switzerland) at 485 nm excitation/535 nm emission for sodium fluorescein and FITC-Dextran 4000, and at 535 nm excitation/590 nm emission for rhodamine B. Apparent permeation coefficients (P_**app**_) were then calculated according to Eq. (1),

1 $${{\boldsymbol{P}}}_{{\boldsymbol{a}}{\boldsymbol{p}}{\boldsymbol{p}}}=\frac{{\boldsymbol{d}}{\boldsymbol{Q}}}{{\boldsymbol{d}}{\boldsymbol{t}}*{{\boldsymbol{c}}}_{0}*{\boldsymbol{A}}}$$

with *dQ/dt* as flux (µg/s), *c*_*0*_ as initial donor concentration (µg/mL) and *A* as surface area (cm^2^).

### Immunofluorescence staining

For immunofluorescence staining, cultures were fixed overnight in 4% formaldehyde (Carl Roth, Germany) and then rinsed three times with PBS. Cells were permeabilized for 10 min in 0.1% Triton X-100 (ICN, USA), washed thoroughly with PBS, then blocked for 1 h in 10% normal goat serum (Vector Laboratories Inc., USA) containing 0.1% Tween 20 in PBS. The anti-acetylated α-tubulin primary antibody (host: rabbit, ab179484, Abcam, UK) was applied at 1:800 dilution in 0.1% BSA/Tris-buffered saline with 0.05% Triton X-100 and incubated overnight at 4 °C. After three PBS washes, samples were exposed for 1 h to a 1:200 dilution of FITC-conjugated anti-rabbit IgG secondary antibody (host: goat, ab6717, Abcam, UK). Nuclei were counterstained with 20 µg/mL Hoechst 33,342 (Sigma-Aldrich, Germany) for 20 min. Following two final PBS washes, stained cultures were imaged using an Olympus IX73 microscope using cellSens software. Raw images were pseudo-colored and adjusted for brightness and contrast in ImageJ (NIH, USA). Immunofluorescence was quantified by calculating the percentage of the image area that was stained, according to the previously described protocol [49].

### Cilia length

Cilia length was assessed weekly during ALI culture as described before [50]. Cells were detached by incubation in 0.02% EDTA (MP Biomedicals, USA) for 5 min, followed by 0.05% trypsin (Thermo Fisher Scientific, USA) for 15 min. Digestion was stopped with soybean trypsin inhibitor (Thermo Fisher Scientific, USA), and cells were pelleted at 1246 RFC for 3 min. Pellets were fixed in 500 µL of 4% formaldehyde (Carl Roth, Japan), mounted on coverslips, and imaged at 20 × magnification on an Olympus IX50 inverted microscope with an Olympus XC30 camera (Olympus, Japan). Cilia length was measured at least ten cilia per sample using cellSens software.

### Statistical analysis

Data for mucin quantification, cilia length and barrier properties are presented as mean ± standard deviation (SD). ANOVA followed up by a Tukey test was performed for statistical analysis. *P*-values less than 0.05 were considered statistically significant. For clarity, statistical significance indicators were excluded from figures. The calculated *p*-values can be found in the supplementary data. Independent biological replicates are denoted by n, while multiple measurements taken from different regions of the same sample are denoted by m.

## Results and discussion

### Histological sections

**Fig. 1:**
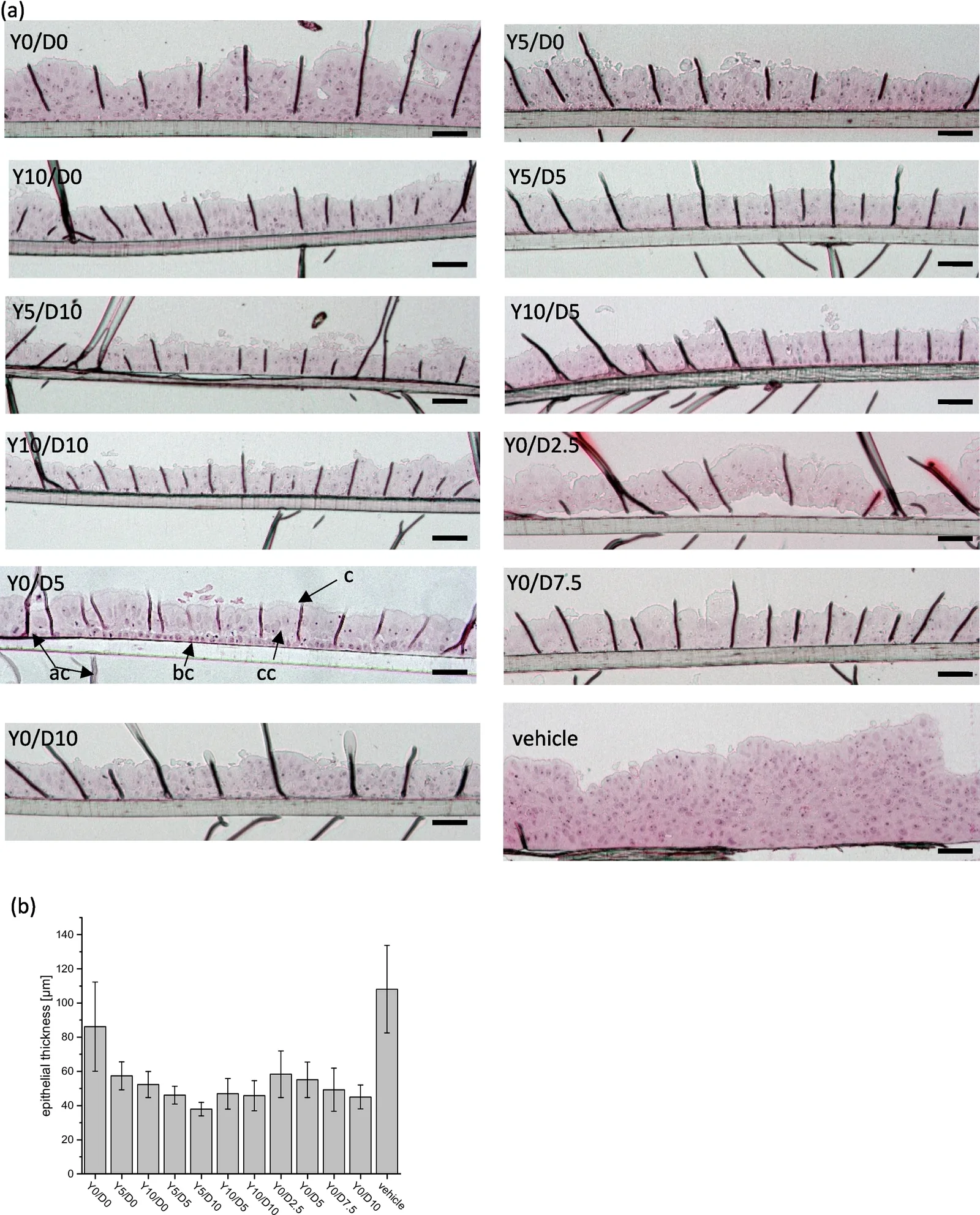
(**a**) Histological sections with subsequent hematoxylin and eosin staining. The Y0/D0 culture expresses an epithelium of inhomogeneous thickness, aberrant from physiological nasal mucosa, with sparse ciliation. Supplementing the culture with Y-27632 led to a homogenization of the histological architecture. Cultures supplemented with DAPT express a pseudostratified epithelium similar to that found in human nasal mucosa. Notably, Y0/D2.5 and Y0/D5 cultures yield multiple cilia. The vehicle control shows a highly altered histological organization, with no visible pseudostratified structure or ciliation. Scale bars correspond to 50 µm. All figures display creases in varying extents as artifacts of the Technovit^®^ fixation, formed by the culture membrane during the cutting process and should therefore not be considered in the evaluation. Exemplary, cilia (c), basal cells (bc), columnar cells (cc) and artificial creases (ac) were labeled in Y0/D5. Sections of ex vivo human nasal epithelium can be found in supplementary Table 1. (**b**) Measured epithelial thickness. Mean ± SD, *m* = 77–138, *p*-values can be found in supplementary Table 2

Cultures with no supplementation displayed a highly heterogeneous histoarchitecture (Fig. 1a). In these non-supplemented cultures, discrete regions present an organotypic pseudostratified epithelium adjacent to disorganized multilayered epithelium, increased cell density, and atypical differentiation can be detected. Vehicle control cultures diverged markedly from native nasal epithelium, displaying a thickened, multilayered epithelium. In contrast, dual supplementation of DAPT and Y-27632 promoted a markedly more uniform epithelial architecture. Treated cultures displayed a continuous, monolayer‐organized basal cell compartment and regularly arrayed columnar cells at the apical surface, thereby precisely reproducing the pseudostratified culture of physiological nasal epithelium. Notably, Y5/D5, Y10/D5 and Y10/D10 consistently achieved an epithelial thickness of 40–50 µm (Fig. 1b). This closely matched the pseudostratified nasal mucosa in vivo and demonstrated a high degree of uniformity in width and cellular organization. Y5/D10 also showed an improved uniformity, but these cultures fell below the physiological thickness of 40 µm. Sections obtained from DAPT‐supplemented cultures frequently showed well‐developed cilia on the apical surface whereas Y-27632 appeared to contribute primarily to maintaining epithelial structure and reducing aberrant cell stratification. When used individually, DAPT alone boosted ciliogenesis but did not fully prevent occasional multilayering in higher concentrations, whereas Y-27632 alone improved monolayer organization. Nonetheless, both supplements improved the epithelial uniformity and reduced the epithelial thickness significantly, while remaining within the physiological benchmark of 40–100 µm.

Both tested pathways are involved in cell overgrowth in a variety of cancer cell lines. In tumors, hyperactivation of these pathways promotes proliferation and invasiveness by promoting mitosis and suppressing apoptosis [34, 51]. It is presumed that the reduction in epithelial thickness in CI-pNaEC follows a similar mechanism to that observed in these cancer cell lines, thereby reducing epithelial overgrowth, as seen in Y0/D0 and the vehicle control.

### Ciliation

#### Immunofluorescence staining

**Fig. 2:**
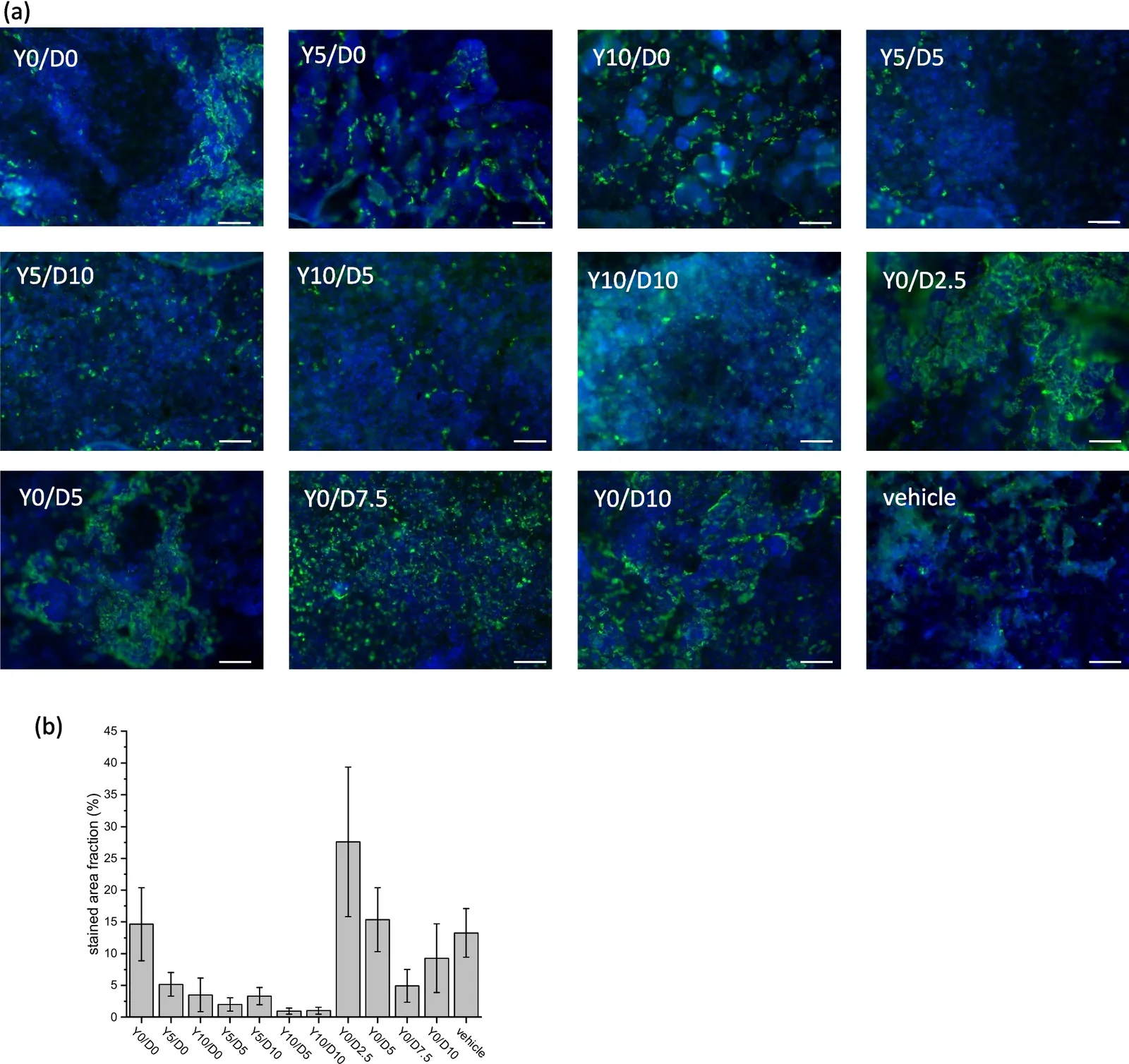
Ciliation in CI-pNaEC cultures. **(a)** Top view of immunofluorescence staining at 10 × magnification. Acetylated α-tubulin is shown in green, cell nuclei in blue. Multiciliated cells are visible in all cultures. Y5/D0 and Y10/D0 show a homogeneous distribution of ciliated cells, while expressing fewer ciliated cells compared to controls. Combination of DAPT and Y-27632 led to an overall decrease in ciliation. Ciliation increased visibly in Y0/D2.5 and Y0/D5 compared toY0/D0 and vehicle controls. Scale bar corresponds to 100 µm. **(b)** Quantification of ciliation by measuring the area fraction stained by α-tubulin. Mean ± SD, *m* = 5–9, *p*-values can be found in supplementary Table 3

Untreated controls expressed a highly heterogeneous ciliation across the epithelial surface (Fig. 2). Certain regions exhibited dense, uniformly distributed cilia, while adjacent regions showed absence of cilia. Vehicle control exhibited no significant reduction in ciliation compared to Y0/D0 culture. Supplementation with Y-27632 alone led to further suppression of ciliogenesis and yielded uniformly low levels of ciliation with reduced spatial variability, which is in accordance with previous findings [46]. By contrast, DAPT supplementation induced a clear, dose-dependent increase in ciliation. In Y0/D2.5 ciliation increased significantly compared to both Y0/D0 and vehicle control. However, higher DAPT doses caused a decline in ciliation. This biphasic, dose-dependent effect is consistent with previous reports showing that moderate γ-secretase inhibition promotes airway epithelial ciliogenesis, whereas stronger inhibition paradoxically reduces ciliation [52–54]. In co-supplemented cultures, ciliation decreased further in an additive manner.

#### Cilia length

**Fig. 3:**
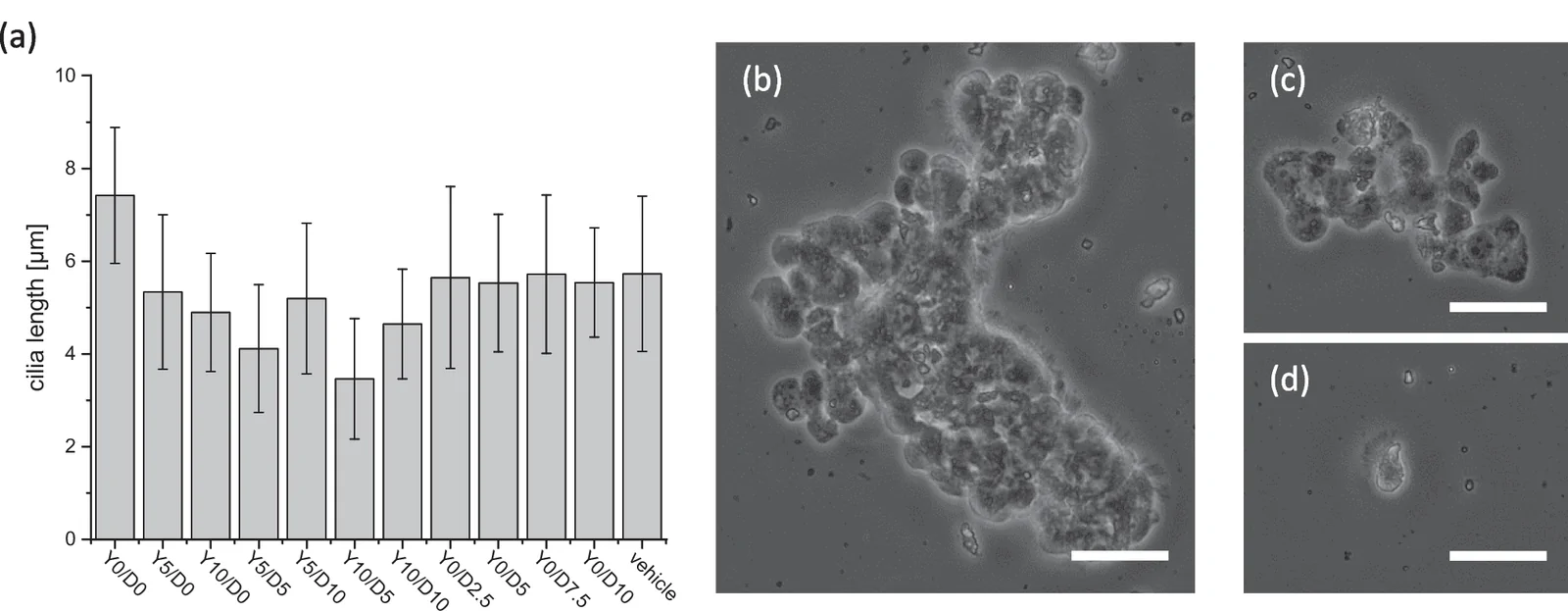
Cilia length in CI-pNaEC cultures after 21 days of ALI cultivation. **(a**) Determined cilia lengths. **(b**) Multiciliated cell cluster of Y0/D0 cultures. Scale bar corresponds to 25 µm. (**c)** Cell cluster of Y10/D5 culture with few ciliated cells. Scale bar corresponds to 25 µm. **(d)** Multiciliated cell of Y0/D2.5 culture. Scale bar corresponds to 25 µm. Mean ± SD, *m* = 39–202, *p*-values can be found in supplementary Table 4

Airway cilia express a physiological length of 5–7 µm [55–57]. In blank controls Y0/D0, cilia expressed a length of 7.4 ± 1.5 µm, slightly exceeding the physiological range (Fig. 3). Addition of the vehicle led to a modest but statistically significant shortening of cilia to 5.7 ± 1.7 µm, effectively normalizing cilia length to the physiological length. Treatment with DAPT alone resulted in a mean cilia length, which did not differ significantly from vehicle control. This indicates that while DAPT robustly increases the number of ciliated cells, it does not further influence individual cilia length beyond the DMSO-induced shortening. In previous studies, a cilia length inducing effect of DAPT was observed in mouse tracheal cells [52], which seems to not apply to CI-pNaEC cultures. This could be a consequence of the immortalization process, which has been described as being capable of influencing cell behavior [58, 59].

Addition of Y-27632 resulted in a significant shortening of cilia length when exposed to higher concentrations compared to vehicle control (Y5/D0 = 5.3 ± 1.7 µm; Y10/D0 = 4.9 ± 1.3 µm). In co-supplemented cultures with high dose DAPT (Y5/D10 = 5.2 ± 1.6 µm; Y10/D10 = 4.6 ± 1.2 µm) ciliary length at both Y-27632 concentrations showed a significant shortening only in high concentrations of Y-27632 compared to the corresponding mono-supplemented DAPT cultures. Furthermore, co-supplemented cultures with lower DAPT concentrations (Y5/D5 = 4.1 ± 1.4 µm; Y10/D5 = 3.5 ± 1.3 µm) led to a further decrease in cilia length compared to Y5/D0 and Y10/D0 cultures, mono-supplemented DAPT cultures, and co-supplemented cultures of high DAPT concentrations.

These results imply, that in addition to ROCK signaling being essential for ciliogenesis by promoting basal‐body docking, its inhibition affects ciliary length in an undescribed manner. Remarkably, co-treatment with the γ-secretase inhibitor DAPT and the ROCK inhibitor Y-27632 results in a cilia-shortening effect at low DAPT concentrations, suggesting a more complex interplay between these pathways in the control of ciliary length.

### Mucus expression

**Fig. 4:**
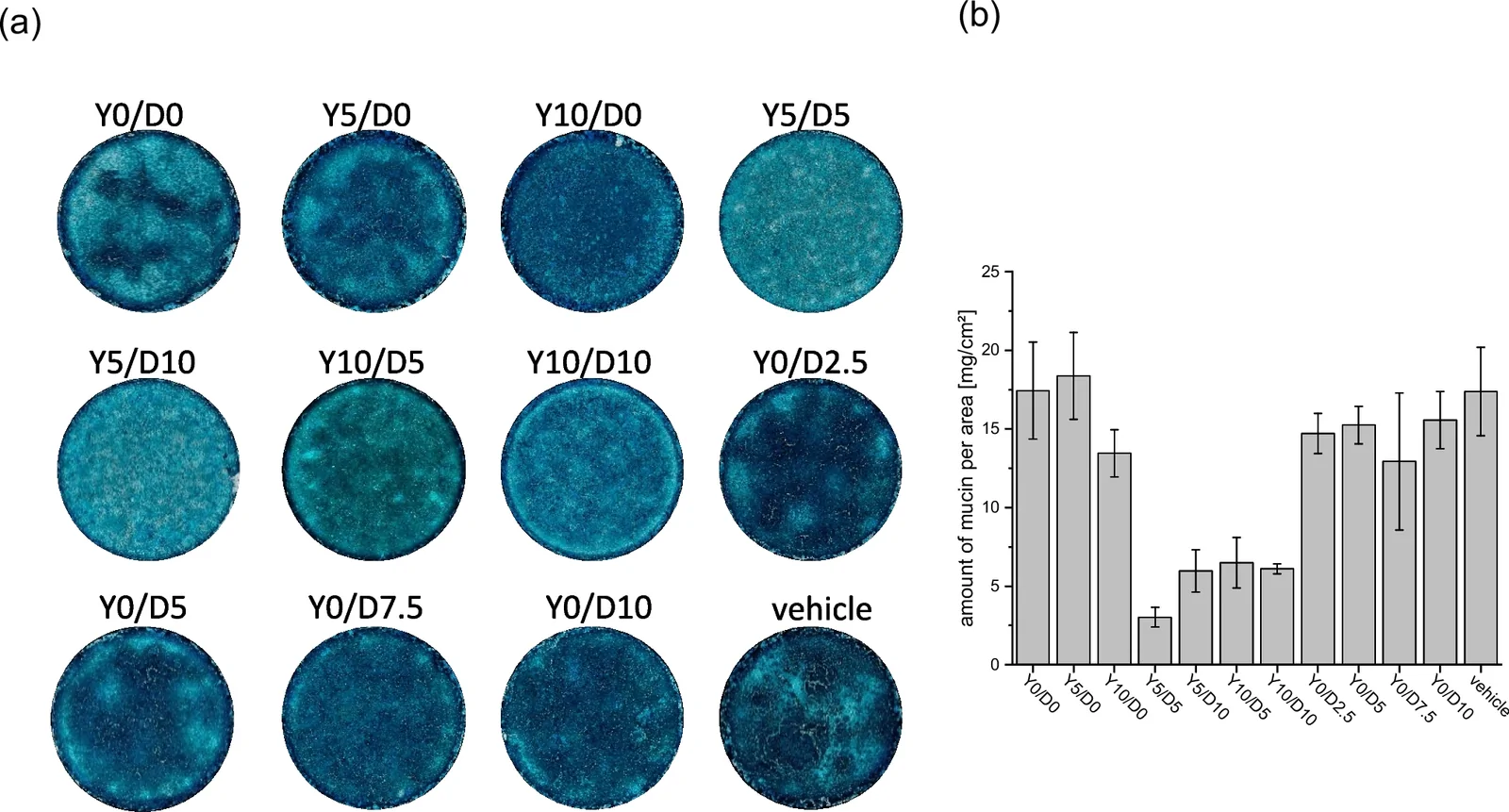
Evaluation of mucus expression. **(a)** Top view of alcian blue stained inserts indicating mucus distribution over the culture. Deeper blue staining implies stronger mucus accumulation. A homogeneous mucus distribution is characterized by a uniform blue staining of the whole culture. **(b)** Mucin quantification in relation to the culture area after 21 days ALI culture. Mean ± SD, *n* = 2–3, *p*-values can be found in supplementary Table 5

Mucus distribution and total secreted mucin were assessed (Fig. 4). Blank control (Y0/D0) cultures exhibited a highly heterogeneous mucus layer, with discrete areas of intense alcian blue staining alternating with regions showing little staining, indicating local mucus accumulation. Quantitative analysis of secreted mucin yielded 17.44 ± 3.08 mg as reference baseline, with no significant variation in vehicle control (17.38 ± 2.81 mg), while vehicle control demonstrated a slight improvement in spatial uniformity of the mucus layer. Treatment with Y-27632 maintained total mucin levels comparable to controls (Y5/D0 = 18.38 ± 2.77 mg; Y10/D0 = 13.46 ± 1.49 mg) but led to a continuous, evenly distributed mucus layer. Mucin expressed in DAPT supplemented cultures did not differ significantly from controls (Y0/D2.5 = 14.72 ± 1.28 mg; Y0/D5 = 15.25 ± 1.20 mg; Y0/D7.5 = 12.94 ± 4.36 mg; Y0/D10 = 15.57 ± 1.82 mg), yet similarly to Y-27632 mucus coverage homogenized compared to controls.

By contrast, the combination of Y-27632 and DAPT significantly reduced total mucin secretion and expressed a thin, uniformly dense mucus layer. This synergistic reduction may reflect combined modulation of cytoskeletal dynamics and Notch-dependent goblet cell differentiation. Remarkably, an effect of ROCK on mucus production was not described before.

Furthermore, variations in ciliation likely contribute to the observed changes in mucus distribution. High cilia density combined with coordinated, metachronal beating facilitates efficient mucociliary transport and can yield a uniform mucus layer. In contrast, regions with sparse or poorly synchronized cilia function as local retention sites, promoting focal mucus accumulation. In addition, missing directional clearance can influence the distribution of mucus. Thus, both the overall density and the spatial coordination of cilia critically determine the pattern of mucus spreading and retention on the epithelial surface. However, this effect was not quantified in this study.

Another limitation of the present study is the absence of mucin subtype analysis. Due to the different physicochemical properties of MUC5AC and MUC5B, future studies should quantify specific mucin species and evaluate mucus rheology. This will help to determine how Y-27632 and DAPT affect mucus viscoelasticity in CI-pNaEC models. Additionally, it is unclear whether the reduction in mucin production is due to a smaller number of goblet cells or impaired secretory activity despite an unchanged goblet cell population.

### Barrier properties

**Fig. 5:**
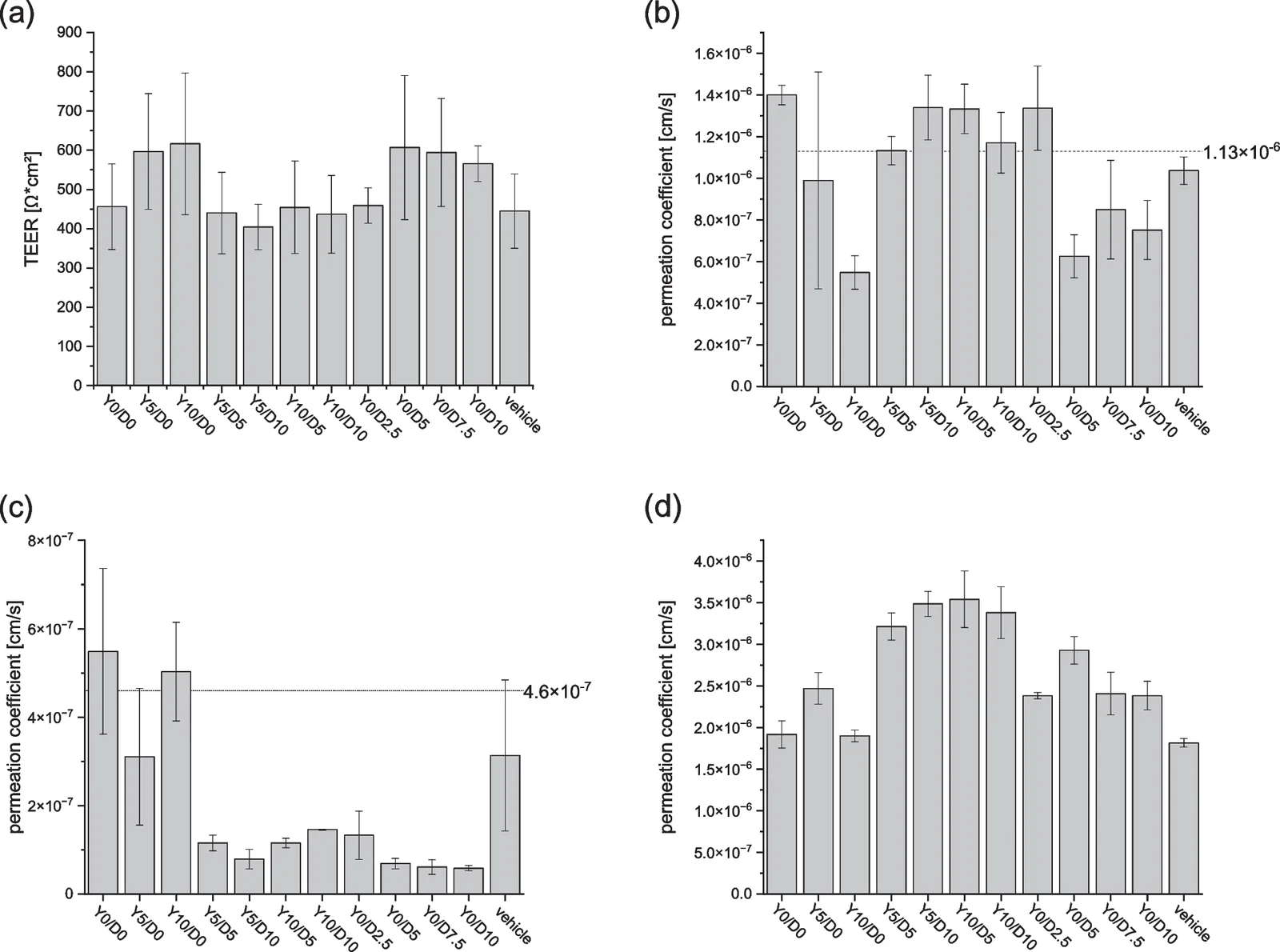
Assessment of the barrier properties after 21 days of ALI culture. (**a)** Comparison of TEER values as a surrogate parameter for the barrier integrity. (**b)** Permeation coefficients of sodium fluorescein as a marker substance for paracellular transport. Lower limit of ex vivo permeation coefficients is marked at 1.13·10^–6^ cm/s [60]. **(c)** Permeation coefficients of FITC-Dextran 4000 as a compound with high molecular weight. Lower limit of ex vivo permeation coefficients is marked at 4.6·10^–7^ cm/s [61–63]. **(d)** P_app_ values of rhodamine B as a lipophilic marker substance. Mean ± SD, *n* = 3–10, *p*-values can be found in supplementary Tables 6–9

The results of permeation experiments are displayed in Fig. 5. Epithelial barrier function was assessed in all culture conditions by measuring TEER and by determining apparent permeation coefficients of the three marker substances sodium fluorescein, FITC-Dextran 4000, and rhodamine B. Non-supplemented cultures (Y0/D0) exhibited a baseline TEER of 456 ± 109 Ω·cm^2^. DMSO vehicle control did not differ significantly from Y0/D0 despite expressing a markedly thickened epithelium (Sect. 3.1). In contrast, supplementation with Y-27632 led to a significant increase in TEER. Furthermore, DAPT supplementation led to significant enhancement at concentrations of 5 and 7.5 µM, while Y0/D2.5 and Y0/D10 did not show a significant influence. Interestingly, co-supplemented cultures did not show a significantly altered TEER compared to controls. However, co-supplemented cultures expressed significantly lower TEER compared to the corresponding mono supplemented cultures for most cultures. This effect was not visible in Y10/D5 cultures compared to Y0/D5.

For paracellular permeability testing using sodium fluorescein, cultures supplemented with Y-27632 and DAPT alone show a concentration dependent trend toward reduced P_app_. In co-supplemented cultures, no visible decrease in permeability is apparent. Studying ex vivo tissue, permeation coefficients range from 1.13·10^–6^ to 5.11·10^–6^ cm/s [60]. Most of the tested media compositions fell within this benchmark range. However, supplementation with DAPT caused a pronounced decrease in permeation rate, resulting in permeation coefficients that no longer matched the target ex vivo P_app_ values.

In FITC-Dextran 4000 permeation experiments representative of high molecular weight drugs, the vehicle control showed no significant deviation in the permeation coefficient compared to Y0/D0 blank control. Supplementation with Y-27632 led to no significant change in the P_app_ value, while co-supplemented cultures exhibited significantly lower permeability. Furthermore, mono-supplementation with DAPT reduced permeability significantly, yet higher dosages did not lead to a further decrease (Y0/D2.5: 1.33 ± 0.55·10^–7^ cm/s; Y0/D5: 6.87 ± 1.19·10^–8^ cm/s; Y0/D7.5: 5.82 ± 0.62 ·10^–8^ cm/s; Y0/D10: 6.08 ± 1.66·10^–8^ cm/s). These reductions in FITC-Dextran 4000 permeability suggest that γ-secretase inhibition imposes a stronger barrier to the paracellular passage of larger solutes. In the case of ex vivo animal nasal mucosa, reported permeation coefficients range from 0.46·10^–6^ to 1.275·10^–6^ cm/s [61–63]. Accordingly, DAPT supplementation in every tested medium formulation and concentration resulted in permeation coefficients below this physiological benchmark.

Notably, neither TEER-development nor permeability of sodium fluorescein and FITC-Dextran 4000 correlated with epithelial thickness. In the nasal mucosa, goblet cells form a weaker barrier than basal and ciliated cells due to a lack in tight junction protein claudin-3 expression [64]. Thus, although the epithelium was thinner in supplemented cultures, shifts in cell-type composition could be related to the increased barrier integrity in mono supplemented cultures. However, cellular proportions were not quantified in this study and effects of ROCK and Notch pathways on barrier integrity were not assessed before. Consequently, further work combining quantitative cell-type analysis with molecular readouts of tight junction components is required to elucidate the mechanisms driving the changes in barrier properties. Despite these limitations, DAPT supplementation clearly enhanced resistance to FITC-Dextran 4000 permeation, indicating a functional improvement in barrier selectivity toward high molecular weight molecules. Furthermore, DMSO vehicle influenced tracer permeability, suggesting that reducing DMSO concentration in future experiments may further improve epithelial integrity towards physiological benchmarks.

In rhodamine B permeation studies, no statistically significant effects of vehicle control can be observed. However, a significant increase in the permeation coefficient is detectable in most supplemented cultures. Permeation of rhodamine B was proven to be highly influenced by the amount of secreted mucus, as rhodamine B in its positively charged structure shows higher interactions to the mucins compared to sodium fluorescein and FITC-Dextran 4000 [29, 65, 66]. Thus, this decrease in the permeation barrier could also be related to the decreased mucin expression as described in Sect. 3.3 for co-supplemented cultures. Furthermore, transcellular permeation properties can be influenced by epithelial thickness [67, 68]. In Sect. 3.1, a distinct variation in the epithelial thickness was explored, demonstrating that vehicle control expressed the thickest epithelium. Correspondingly, vehicle control expressed the lowest permeation coefficient.

Overall, addition of both DAPT and Y-27632 seems to influence the permeability of the tested mucosa models. To clarify the basis for these divergent effects, future studies should aim to explain the underlying mechanisms of DAPT-mediated Notch inhibition and Y-27632 induced ROCK inhibition on the formation of the permeation barrier for example as an influence on the tight junction protein expression. 

## Conclusion

The influence of DAPT and Y-27632 supplementation on the differentiation of in vitro models based on the cell line CI-pNaEC was evaluated. Both supplements modulated key differentiation endpoints. However, the most pronounced improvements were observed in cultures treated with 2.5 µM DAPT in the absence of Y-27632. Under these conditions, the epithelium developed a more uniform pseudostratified architecture, exhibited significantly increased ciliation and a physiological cilia length, and achieved more homogeneous mucus distribution, all without any significant decline in mucin expression. However, DAPT supplementation also led to a dose dependent tightening of the epithelial barrier, which fell below target ex vivo benchmarks. Therefore, DAPT supplementation may enhance mucociliary clearance by promoting ciliogenesis and increasing the number of functional cilia and thereby improve the quality of the in vitro model.

The previously reported inhibitory effect of Y-27632 on ciliation was confirmed in this study, yet supplementation with this ROCK inhibitor improved tissue histological architecture. Interestingly, the addition of 10 µM Y-27632 induced cilia shortening, an effect not previously described. Furthermore, the combination of both supplements led to an improvement in histology, decreased ciliation and mucin expression and only a slight modulation of the permeability.

Future work should refine DAPT and Y-27632 dosing regimens and reduced DMSO vehicle dosages, both in terms of concentration and exposure duration, in order to optimize barrier tightening without forfeiting epithelial differentiation. In addition, more complex treatments consisting of transient exposure to DAPT and Y-27632 could be explored. Furthermore, integrating dynamic culture platforms such as organ-on-a-chip devices offer the potential to further improve the quality of the in vitro model [69].

## Supplementary Information

Below is the link to the electronic supplementary material.

Supplementary file1 (DOCX 929 KB)

## Data Availability

Raw data are available from the corresponding author upon reasonable request.
