# Supplementary material for: Influence of Notch inhibitor DAPT and ROCK inhibitor Y-27632 on differentiation in nasal cell line CI-pNaEC as an in vitro model for preclinical testing of drugs
Source: In Vitro Model. 2026 Aug 19;5(4):737–49. doi: 10.1007/s44164-026-00145-7 (PMC13768446; doi:10.1007/s44164-026-00145-7)
Supplement: Supplementary file 1 [file 44164_2026_145_MOESM1_ESM.docx]

Supplementary data

| Feature | Physiological characteristics | References |
| --- | --- | --- |
| Histology |   Histological section of human nasal epithelium adapted from Dolberg. Scale bar corresponds to 100 µm.  Thickness of 40-100 µm (Beule, 2010)  42-78 µm (Hadar et al., 2009)  61.5-81.5 µm (Schrödter et al., 2003) | Beule, A. G. (2010). Physiology and Pathophysiology of Respiratory Mucosa of the Nose and the Paranasal Sinuses. *GMS Current Topics in Otorhinolaryngology, Head and Neck Surgery*, *9*, Doc07. https://doi.org/10.3205/cto000071  Dolberg, A. M. (2021) *Expression and activity of ABC and SLC transporters in differently cultured RPMI 2650 cells and specimens of human nasal mucosa.* Dissertation, TU Braunschweig  Gizurarson, S. (2012). Anatomical and histological factors affecting intranasal drug and vaccine delivery. *Current Drug Delivery*, *9*(6), 566–582. https://doi.org/10.2174/156720112803529828  Gudis, D. A., & Cohen, N. A. (2010). Cilia dysfunction. *Otolaryngologic Clinics of North America*, *43*(3), 461-72, vii. https://doi.org/10.1016/j.otc.2010.02.007  Hadar, T., Yaniv, E., Shvili, Y., Koren, R., & Shvero, J. (2009). Histopathological changes of the nasal mucosa induced by smoking. *Inhalation Toxicology*, *21*(13), 1119–1122. https://doi.org/10.3109/08958370902767070  Schrödter, S., Biermann, E., & Halata, Z. (2003). Histological evaluation of age-related changes in human respiratory mucosa of the middle turbinate. *Anatomy and Embryology*, *207*(1), 19–27. https://doi.org/10.1007/s00429-003-0326-5 |
| Cilia length | 5 µm (Gizurarson, 2012)  6 µm (Beule, 2010)  5-7 µm (Gudis & Cohen, 2010) |  |

**Supplementary table 1.** Overview of physiological benchmarks

|  | Y0/D0 | Y5/D0 | Y10/D0 | Y5/D5 | Y5/D10 | Y10/D5 | Y10/D10 | Y0/D2.5 | Y0/D5 | Y0/D7.5 | Y0/D10 | vehicle |
| --- | --- | --- | --- | --- | --- | --- | --- | --- | --- | --- | --- | --- |
| Y0/D0 |  | 6.04E-20 | 6.91E-27 | 2.36E-38 | 1.24E-43 | 1.83E-35 | 3.17E-36 | 5.26E-16 | 3.71E-24 | 9.44E-27 | 4.69E-38 | 9.41E-09 |
| Y5/D0 | 6.04E-20 |  | 5.04E-06 | 3.35E-27 | 4.69E-55 | 6.03E-17 | 7.46E-20 | 0.5910 | 0.0631 | 1.20E-07 | 4.63E-26 | 9.25E-50 |
| Y10/D0 | 6.91E-27 | 5.04E-06 |  | 7.83E-12 | 8.14E-42 | 1.97E-06 | 1.25E-08 | 1.19E-04 | 0.0263 | 0.0341 | 1.57E-12 | 3.74E-59 |
| Y5/D5 | 2.36E-38 | 3.35E-27 | 7.83E-12 |  | 2.79E-29 | 0.3873 | 0.7572 | 1.08E-16 | 4.44E-15 | 0.0127 | 0.1744 | 5.46E-74 |
| Y5/D10 | 1.24E-43 | 4.69E-55 | 8.14E-42 | 2.79E-29 |  | 2.90E-18 | 3.98E-15 | 2.79E-33 | 4.35E-38 | 6.79E-16 | 4.11E-17 | 9.85E-77 |
| Y10/D5 | 1.83E-35 | 6.03E-17 | 1.97E-06 | 0.3873 | 2.90E-18 |  | 0.3324 | 2.51E-12 | 4.20E-10 | 0.1027 | 0.0673 | 1.44E-71 |
| Y10/D10 | 3.17E-36 | 7.46E-20 | 1.25E-08 | 0.7572 | 3.98E-15 | 0.3324 |  | 3.51E-14 | 2.38E-12 | 0.0170 | 0.4387 | 7.18E-72 |
| Y0/D2.5 | 5.26E-16 | 0.5910 | 1.19E-04 | 1.08E-16 | 2.79E-33 | 2.51E-12 | 3.51E-14 |  | 0.0488 | 2.17E-06 | 3.39E-17 | 2.02E-43 |
| Y0/D5 | 3.71E-24 | 0.0631 | 0.0263 | 4.44E-15 | 4.35E-38 | 4.20E-10 | 2.38E-12 | 0.0488 |  | 2.16E-04 | 6.28E-16 | 5.02E-58 |
| Y0/D7.5 | 9.44E-27 | 1.20E-07 | 0.0341 | 0.0127 | 6.79E-16 | 0.1027 | 0.0170 | 2.17E-06 | 2.16E-04 |  | 0.0018 | 8.84E-59 |
| Y0/D10 | 4.69E-38 | 4.63E-26 | 1.57E-12 | 0.1744 | 4.11E-17 | 0.0673 | 0.4387 | 3.39E-17 | 6.28E-16 | 0.0018 |  | 2.07E-73 |
| vehicle | 9.41E-09 | 9.25E-50 | 3.74E-59 | 5.46E-74 | 9.85E-77 | 1.44E-71 | 7.18E-72 | 2.02E-43 | 5.02E-58 | 8.84E-59 | 2.07E-73 |  |

**Supplementary table 2.** Pairwise p-values of epithelial thickness after 21 days ALI shown in Fig. 1. red - p < 0.05; blue - p < 0.01; green - p < 0.001

|  | Y0/D0 | Y5/D0 | Y10/D0 | Y5/D5 | Y5/D10 | Y10/D5 | Y10/D10 | Y0/D2.5 | Y0/D5 | Y0/D7.5 | Y0/D10 | vehicle |
| --- | --- | --- | --- | --- | --- | --- | --- | --- | --- | --- | --- | --- |
| Y0/D0 |  | 0.0033 | 7.70E-04 | 3.54E-04 | 1.51E-04 | 5.83E-05 | 7.16E-06 | 0.0402 | 0.8184 | 0.0020 | 0.1119 | 0.6618 |
| Y5/D0 | 0.0033 |  | 0.2287 | 0.0047 | 0.0509 | 1.25E-04 | 2.51E-05 | 0.0012 | 6.97E-04 | 0.8615 | 0.1059 | 0.0013 |
| Y10/D0 | 7.70E-04 | 0.2287 |  | 0.2206 | 0.8455 | 0.0282 | 0.0153 | 3.34E-04 | 1.37E-04 | 0.3278 | 0.0266 | 3.80E-04 |
| Y5/D5 | 3.54E-04 | 0.0047 | 0.2206 |  | 0.0770 | 0.0360 | 0.0313 | 4.56E-04 | 5.58E-05 | 0.0247 | 0.0082 | 6.57E-05 |
| Y5/D10 | 1.51E-04 | 0.0509 | 0.8455 | 0.0770 |  | 8.92E-04 | 3.34E-04 | 9.78E-05 | 1.92E-05 | 0.1401 | 0.0097 | 2.79E-05 |
| Y10/D5 | 5.83E-05 | 1.25E-04 | 0.0282 | 0.0360 | 8.92E-04 |  | 0.8338 | 1.15E-04 | 7.07E-06 | 0.0017 | 0.0016 | 6.46E-06 |
| Y10/D10 | 7.16E-06 | 2.51E-05 | 0.0153 | 0.0313 | 3.34E-04 | 0.8338 |  | 1.44E-05 | 6.27E-07 | 5.25E-04 | 4.23E-04 | 4.86E-07 |
| Y0/D2.5 | 0.0402 | 0.0012 | 3.34E-04 | 4.56E-04 | 9.78E-05 | 1.15E-04 | 1.44E-05 |  | 0.0318 | 5.20E-04 | 0.0044 | 0.0321 |
| Y0/D5 | 0.8184 | 6.97E-04 | 1.37E-04 | 5.58E-05 | 1.92E-05 | 7.07E-06 | 6.27E-07 | 0.0318 |  | 3.88E-04 | 0.0509 | 0.4589 |
| Y0/D7.5 | 0.0020 | 0.8615 | 0.3278 | 0.0247 | 0.1401 | 0.0017 | 5.25E-04 | 5.20E-04 | 3.88E-04 |  | 0.0799 | 0.0011 |
| Y0/D10 | 0.1119 | 0.1059 | 0.0266 | 0.0082 | 0.0097 | 0.0016 | 4.23E-04 | 0.0044 | 0.0509 | 0.0799 |  | 0.1898 |
| vehicle | 0.6618 | 0.0013 | 3.80E-04 | 6.57E-05 | 2.79E-05 | 6.46E-06 | 4.86E-07 | 0.0321 | 0.4589 | 0.0011 | 0.1898 |  |

**Supplementary table 3.** Pairwise p-values of immunofluorescence-stained area fraction after 21 days ALI shown in Fig. 2. red - p < 0.05; blue - p < 0.01; green - p < 0.001

|  | Y0/D0 | Y5/D0 | Y10/D0 | Y5/D5 | Y5/D10 | Y10/D5 | Y10/D10 | Y0/D2.5 | Y0/D5 | Y0/D7.5 | Y0/D10 | vehicle |
| --- | --- | --- | --- | --- | --- | --- | --- | --- | --- | --- | --- | --- |
| Y0/D0 |  | 3.22E-15 | 2.18E-20 | 1.76E-36 | 3.69E-19 | 3.01E-29 | 1.33E-18 | 3.88E-11 | 3.04E-20 | 4.54E-11 | 1.18E-15 | 2.46E-11 |
| Y5/D0 | 3.22E-15 |  | 0.08674 | 1.67E-07 | 0.55512 | 4.53E-09 | 0.02110 | 0.24121 | 0.32983 | 0.13958 | 0.37824 | 0.12429 |
| Y10/D0 | 2.18E-20 | 0.08674 |  | 4.90E-04 | 0.21509 | 2.88E-07 | 0.32455 | 0.00793 | 0.00284 | 0.00180 | 0.00267 | 0.00131 |
| Y5/D5 | 1.76E-36 | 1.67E-07 | 4.90E-04 |  | 5.18E-07 | 0.01039 | 0.03769 | 5.33E-10 | 2.59E-14 | 1.75E-11 | 0.00000 | 8.57E-12 |
| Y5/D10 | 3.69E-19 | 0.55512 | 0.21509 | 5.18E-07 |  | 7.11E-09 | 0.05288 | 0.06252 | 0.06561 | 0.02904 | 0.11570 | 0.02396 |
| Y10/D5 | 3.01E-29 | 4.53E-09 | 2.88E-07 | 0.01039 | 7.11E-09 |  | 6.20E-05 | 6.45E-10 | 7.32E-15 | 1.07E-11 | 2.12E-14 | 3.78E-12 |
| Y10/D10 | 1.33E-18 | 0.02110 | 0.32455 | 0.03769 | 0.05288 | 6.20E-05 |  | 0.00306 | 4.99E-04 | 5.15E-04 | 1.90E-04 | 3.61E-04 |
| Y0/D2.5 | 3.88E-11 | 0.24121 | 0.00793 | 5.33E-10 | 0.06252 | 6.45E-10 | 0.00306 |  | 0.54948 | 0.79189 | 0.66768 | 0.76440 |
| Y0/D5 | 3.04E-20 | 0.32983 | 0.00284 | 2.59E-14 | 0.06561 | 7.32E-15 | 4.99E-04 | 0.54948 |  | 0.34531 | 0.96058 | 0.31619 |
| Y0/D7.5 | 4.54E-11 | 0.13958 | 0.00180 | 1.75E-11 | 0.02904 | 1.07E-11 | 5.15E-04 | 0.79189 | 0.34531 |  | 0.44591 | 0.97464 |
| Y0/D10 | 1.18E-15 | 0.37824 | 0.00267 | 1.75E-11 | 0.11570 | 2.12E-14 | 1.90E-04 | 0.66768 | 0.96058 | 0.44591 |  | 0.41657 |
| vehicle | 2.46E-11 | 0.12429 | 0.00131 | 8.57E-12 | 0.02396 | 3.78E-12 | 3.61E-04 | 0.76440 | 0.31619 | 0.97464 | 0.41657 |  |

**Supplementary table 4.** Pairwise p-values of cilia length after 21 days ALI shown in Fig. 3. red - p < 0.05; blue - p < 0.01; green - p < 0.001

|  | Y0/D0 | Y5/D0 | Y10/D0 | Y5/D5 | Y5/D10 | Y10/D5 | Y10/D10 | Y0/D2.5 | Y0/D5 | Y0/D7.5 | Y0/D10 | vehicle |
| --- | --- | --- | --- | --- | --- | --- | --- | --- | --- | --- | --- | --- |
| Y0/D0 |  | 0.7645 | 0.1755 | 0.0029 | 0.0067 | 0.0112 | 0.0277 | 0.3126 | 0.4021 | 0.3722 | 0.4999 | 0.9839 |
| Y5/D0 | 0.7645 |  | 0.0917 | 0.0016 | 0.0037 | 0.0063 | 0.0169 | 0.1650 | 0.2167 | 0.2784 | 0.2961 | 0.7375 |
| Y10/D0 | 0.1755 | 0.0917 |  | 0.0008 | 0.0043 | 0.0109 | 0.0131 | 0.4180 | 0.2570 | 0.8932 | 0.2740 | 0.1567 |
| Y5/D5 | 0.0029 | 0.0016 | 0.0008 |  | 0.1078 | 0.0460 | 0.0150 | 0.0003 | 0.0002 | 0.0574 | 0.0008 | 0.0021 |
| Y5/D10 | 0.0067 | 0.0037 | 0.0043 | 0.1078 |  | 0.4230 | 0.5113 | 0.0019 | 0.0014 | 0.1114 | 0.0029 | 0.0052 |
| Y10/D5 | 0.0112 | 0.0063 | 0.0109 | 0.0460 | 0.4230 |  | 0.8085 | 0.0048 | 0.0035 | 0.1681 | 0.0061 | 0.0089 |
| Y10/D10 | 0.0277 | 0.0169 | 0.0131 | 0.0150 | 0.5113 | 0.8085 |  | 0.0055 | 0.0039 | 0.2582 | 0.0110 | 0.0221 |
| Y0/D2.5 | 0.3126 | 0.1650 | 0.4180 | 0.0003 | 0.0019 | 0.0048 | 0.0055 |  | 0.6890 | 0.6432 | 0.6172 | 0.2900 |
| Y0/D5 | 0.4021 | 0.2167 | 0.2570 | 0.0002 | 0.0014 | 0.0035 | 0.0039 | 0.6890 |  | 0.5492 | 0.8475 | 0.3808 |
| Y0/D7.5 | 0.3722 | 0.2784 | 0.8932 | 0.0574 | 0.1114 | 0.1681 | 0.2582 | 0.6432 | 0.5492 |  | 0.5240 | 0.3626 |
| Y0/D10 | 0.4999 | 0.2961 | 0.2740 | 0.0008 | 0.0029 | 0.0061 | 0.0110 | 0.6172 | 0.8475 | 0.5240 |  | 0.4867 |
| vehicle | 0.9839 | 0.7375 | 0.1567 | 0.0021 | 0.0052 | 0.0089 | 0.0221 | 0.2900 | 0.3808 | 0.3626 | 0.4867 |  |

**Supplementary table 5.** Pairwise p-values of mucin expression after 21 days ALI shown in Fig. 4. red - p < 0.05; blue - p < 0.01; green - p < 0.001

|  | Y0/D0 | Y5/D0 | Y10/D0 | Y5/D5 | Y5/D10 | Y10/D5 | Y10/D10 | Y0/D2.5 | Y0/D5 | Y0/D7.5 | Y0/D10 | vehicle |
| --- | --- | --- | --- | --- | --- | --- | --- | --- | --- | --- | --- | --- |
| Y0/D0 |  | 0.0289 | 0.0295 | 0.7497 | 0.2241 | 0.9778 | 0.6971 | 0.9396 | 0.0414 | 0.0259 | 0.0744 | 0.8089 |
| Y5/D0 | 0.0289 |  | 0.8054 | 0.0189 | 0.0022 | 0.0377 | 0.0157 | 0.0162 | 0.9005 | 0.9694 | 0.6511 | 0.0148 |
| Y10/D0 | 0.0295 | 0.8054 |  | 0.0217 | 0.0040 | 0.0384 | 0.0186 | 0.0217 | 0.9135 | 0.7742 | 0.5153 | 0.0171 |
| Y5/D5 | 0.7497 | 0.0189 | 0.0217 |  | 0.3831 | 0.7863 | 0.9490 | 0.6234 | 0.0305 | 0.0163 | 0.0478 | 0.9189 |
| Y5/D10 | 0.2241 | 0.0022 | 0.0040 | 0.3831 |  | 0.2695 | 0.4086 | 0.0409 | 0.0061 | 0.0015 | 0.0061 | 0.2846 |
| Y10/D5 | 0.9778 | 0.0377 | 0.0384 | 0.7863 | 0.2695 |  | 0.7367 | 0.9164 | 0.0523 | 0.0341 | 0.0892 | 0.8440 |
| Y10/D10 | 0.6971 | 0.0157 | 0.0186 | 0.9490 | 0.4086 | 0.7367 |  | 0.5524 | 0.0265 | 0.0132 | 0.0402 | 0.8623 |
| Y0/D2.5 | 0.9396 | 0.0162 | 0.0217 | 0.6234 | 0.0409 | 0.9164 | 0.5524 |  | 0.0321 | 0.0127 | 0.0470 | 0.6865 |
| Y0/D5 | 0.0414 | 0.9005 | 0.9135 | 0.0305 | 0.0061 | 0.0523 | 0.0265 | 0.0321 |  | 0.8714 | 0.5999 | 0.0249 |
| Y0/D7.5 | 0.0259 | 0.9694 | 0.7742 | 0.0163 | 0.0015 | 0.0341 | 0.0132 | 0.0127 | 0.8714 |  | 0.6678 | 0.0126 |
| Y0/D10 | 0.0744 | 0.6511 | 0.5153 | 0.0478 | 0.0061 | 0.0892 | 0.0402 | 0.0470 | 0.5999 | 0.6678 |  | 0.0411 |
| Vehicle | 0.8089 | 0.0148 | 0.0171 | 0.9189 | 0.2846 | 0.8440 | 0.8623 | 0.6865 | 0.0249 | 0.0126 | 0.0411 |  |

**Supplementary table 6.** Pairwise p-values of TEER after 21 days ALI shown in Fig. 5a. red - p < 0.05; blue - p < 0.01; green - p < 0.001

|  | Y0/D0 | Y5/D0 | Y10/D0 | Y5/D5 | Y5/D10 | Y10/D5 | Y10/D10 | Y0/D2.5 | Y0/D5 | Y0/D7.5 | Y0/D10 | vehicle |
| --- | --- | --- | --- | --- | --- | --- | --- | --- | --- | --- | --- | --- |
| Y0/D0 |  | 0.3297 | 0.0002 | 0.0101 | 0.6280 | 0.4992 | 0.1020 | 0.6862 | 0.0006 | 0.0322 | 0.0035 | 0.4892 |
| Y5/D0 | 0.3297 |  | 0.3010 | 0.7198 | 0.4142 | 0.4149 | 0.6615 | 0.4304 | 0.3868 | 0.7467 | 0.5656 | 0.3733 |
| Y10/D0 | 0.0002 | 0.3010 |  | 0.0014 | 0.0031 | 0.0015 | 0.0062 | 0.0069 | 0.4484 | 0.1628 | 0.1521 | 0.2573 |
| Y5/D5 | 0.0101 | 0.7198 | 0.0014 |  | 0.1599 | 0.1067 | 0.7583 | 0.2497 | 0.0044 | 0.1796 | 0.0263 | 0.4023 |
| Y5/D10 | 0.6280 | 0.4142 | 0.0031 | 0.1599 |  | 0.9641 | 0.3250 | 0.9847 | 0.0057 | 0.0708 | 0.0167 | 0.4694 |
| Y10/D5 | 0.4992 | 0.4149 | 0.0015 | 0.1067 | 0.9641 |  | 0.2886 | 0.9867 | 0.0031 | 0.0613 | 0.0112 | 0.4667 |
| Y10/D10 | 0.1020 | 0.6615 | 0.0062 | 0.7583 | 0.3250 | 0.2886 |  | 0.4015 | 0.0126 | 0.1787 | 0.0435 | 0.4145 |
| Y0/D2.5 | 0.6862 | 0.4304 | 0.0069 | 0.2497 | 0.9847 | 0.9867 | 0.4015 |  | 0.0115 | 0.0920 | 0.0286 | 0.4689 |
| Y0/D5 | 0.0006 | 0.3868 | 0.4484 | 0.0044 | 0.0057 | 0.0031 | 0.0126 | 0.0115 |  | 0.2869 | 0.3681 | 0.2735 |
| Y0/D7.5 | 0.0322 | 0.7467 | 0.1628 | 0.1796 | 0.0708 | 0.0613 | 0.1787 | 0.0920 | 0.2869 |  | 0.6396 | 0.3271 |
| Y0/D10 | 0.0035 | 0.5656 | 0.1521 | 0.0263 | 0.0167 | 0.0112 | 0.0435 | 0.0286 | 0.3681 | 0.6396 |  | 0.3017 |
| Vehicle | 0.4892 | 0.3733 | 0.2573 | 0.4023 | 0.4694 | 0.4667 | 0.4145 | 0.4689 | 0.2735 | 0.3271 | 0.3017 |  |

**Supplementary table 7.** Pairwise p-values of sodium fluorescein permeation coefficients after 21 days ALI shown in Fig. 5b. red - p < 0.05; blue - p < 0.01; green - p < 0.001

|  | Y0/D0 | Y5/D0 | Y10/D0 | Y5/D5 | Y5/D10 | Y10/D5 | Y10/D10 | Y0/D2.5 | Y0/D5 | Y0/D7.5 | Y0/D10 | vehicle |
| --- | --- | --- | --- | --- | --- | --- | --- | --- | --- | --- | --- | --- |
| Y0/D0 |  | 0.1085 | 0.4268 | 0.0135 | 0.0096 | 0.0134 | 0.0180 | 0.0176 | 0.0086 | 0.0080 | 0.0078 | 0.1296 |
| Y5/D0 | 0.1085 |  | 0.3529 | 0.1609 | 0.1049 | 0.1592 | 0.2299 | 0.2247 | 0.0906 | 0.0835 | 0.0797 | 0.8213 |
| Y10/D0 | 0.4268 | 0.3529 |  | 0.0385 | 0.0278 | 0.0380 | 0.0507 | 0.0542 | 0.0246 | 0.0232 | 0.0223 | 0.4731 |
| Y5/D5 | 0.0135 | 0.1609 | 0.0385 |  | 0.1419 | 0.9985 | 0.0730 | 0.6932 | 0.0351 | 0.0326 | 0.0120 | 0.1529 |
| Y5/D10 | 0.0096 | 0.1049 | 0.0278 | 0.1419 |  | 0.1056 | 0.0132 | 0.2684 | 0.5862 | 0.4013 | 0.2657 | 0.1032 |
| Y10/D5 | 0.0134 | 0.1592 | 0.0380 | 0.9985 | 0.1056 |  | 0.0187 | 0.6844 | 0.0150 | 0.0178 | 0.0031 | 0.1520 |
| Y10/D10 | 0.0180 | 0.2299 | 0.0507 | 0.0730 | 0.0132 | 0.0187 |  | 0.7585 | 0.0008 | 0.0020 | 4.12E-05 | 0.2110 |
| Y0/D2.5 | 0.0176 | 0.2247 | 0.0542 | 0.6932 | 0.2684 | 0.6844 | 0.7585 |  | 0.1811 | 0.1501 | 0.1283 | 0.1971 |
| Y0/D5 | 0.0086 | 0.0906 | 0.0246 | 0.0351 | 0.5862 | 0.0150 | 0.0008 | 0.1811 |  | 0.6155 | 0.3300 | 0.0910 |
| Y0/D7.5 | 0.0080 | 0.0835 | 0.0232 | 0.0326 | 0.4013 | 0.0178 | 0.0020 | 0.1501 | 0.6155 |  | 0.8419 | 0.0840 |
| Y0/D10 | 0.0078 | 0.0797 | 0.0223 | 0.0120 | 0.2657 | 0.0031 | 4.12E-05 | 0.1283 | 0.3300 | 0.8419 |  | 0.0810 |
| Vehicle | 0.1296 | 0.8213 | 0.4731 | 0.1529 | 0.1032 | 0.1520 | 0.2110 | 0.1971 | 0.0910 | 0.0840 | 0.0810 |  |

**Supplementary table 8.** Pairwise p-values of FTIC 4000 permeation coefficients after 21 days ALI shown in Fig. 5c. red - p < 0.05; blue - p < 0.01; green - p < 0.001

|  | Y0/D0 | Y5/D0 | Y10/D0 | Y5/D5 | Y5/D10 | Y10/D5 | Y10/D10 | Y0/D2.5 | Y0/D5 | Y0/D7.5 | Y0/D10 | vehicle |
| --- | --- | --- | --- | --- | --- | --- | --- | --- | --- | --- | --- | --- |
| Y0/D0 |  | 0.0354 | 0.8953 | 0.0014 | 0.0006 | 0.0037 | 0.0041 | 0.0170 | 0.0035 | 0.0845 | 0.0492 | 0.4527 |
| Y5/D0 | 0.0354 |  | 0.0164 | 0.0137 | 0.0040 | 0.0178 | 0.0242 | 0.5550 | 0.0621 | 0.7962 | 0.6568 | 0.0094 |
| Y10/D0 | 0.8953 | 0.0164 |  | 0.0005 | 0.0002 | 0.0026 | 0.0028 | 0.0011 | 0.0013 | 0.0542 | 0.0210 | 0.2529 |
| Y5/D5 | 0.0014 | 0.0137 | 0.0005 |  | 0.1563 | 0.2875 | 0.5386 | 0.0021 | 0.1545 | 0.0200 | 0.0077 | 0.0003 |
| Y5/D10 | 0.0006 | 0.0040 | 0.0002 | 0.1563 |  | 0.8460 | 0.6864 | 0.0005 | 0.0237 | 0.0068 | 0.0024 | 0.0001 |
| Y10/D5 | 0.0037 | 0.0178 | 0.0026 | 0.2875 | 0.8460 |  | 0.6483 | 0.0087 | 0.0833 | 0.0198 | 0.0127 | 0.0021 |
| Y10/D10 | 0.0041 | 0.0242 | 0.0028 | 0.5386 | 0.6864 | 0.6483 |  | 0.0107 | 0.1422 | 0.0270 | 0.0165 | 0.0022 |
| Y0/D2.5 | 0.0170 | 0.5550 | 0.0011 | 0.0021 | 0.0005 | 0.0087 | 0.0107 |  | 0.0103 | 0.8949 | 0.9911 | 0.0002 |
| Y0/D5 | 0.0035 | 0.0621 | 0.0013 | 0.1545 | 0.0237 | 0.0833 | 0.1422 | 0.0103 |  | 0.0739 | 0.0318 | 0.0008 |
| Y0/D7.5 | 0.0845 | 0.7962 | 0.0542 | 0.0200 | 0.0068 | 0.0198 | 0.0270 | 0.8949 | 0.0739 |  | 0.9165 | 0.0332 |
| Y0/D10 | 0.0492 | 0.6568 | 0.0210 | 0.0077 | 0.0024 | 0.0127 | 0.0165 | 0.9911 | 0.0318 | 0.9165 |  | 0.0110 |
| Vehicle | 0.4527 | 0.0094 | 0.2529 | 0.0003 | 0.0001 | 0.0021 | 0.0022 | 0.0002 | 0.0008 | 0.0332 | 0.0110 |  |

**Supplementary table 9**. Pairwise p-values of rhodamine B permeation coefficients after 21 days shown in Fig. 5d. red - p < 0.05; blue - p < 0.01; green - p < 0.001
